# Oral Microbiota and Salivary Levels of Oral Pathogens in Gastro-Intestinal Diseases: Current Knowledge and Exploratory Study

**DOI:** 10.3390/microorganisms9051064

**Published:** 2021-05-14

**Authors:** Maria Contaldo, Alessandra Fusco, Paola Stiuso, Stefania Lama, Antonietta Gerarda Gravina, Annalisa Itro, Alessandro Federico, Angelo Itro, Gianna Dipalma, Francesco Inchingolo, Rosario Serpico, Giovanna Donnarumma

**Affiliations:** 1 Multidisciplinary Department of Medical-Surgical and Dental Specialties, University of Campania Luigi Vanvitelli, Via Luigi de Crecchio, 6, 80138 Naples, Italy; angelo.itro@unicampania.it; 2 Department of Experimental Medicine, University of Campania Luigi Vanvitelli, Via Luigi de Crecchio, 8, 80138 Naples, Italy; alessandra.fusco@unicampania.it (A.F.); giovanna.donnarumma@unicampania.it (G.D.); 3 Department of Precision Medicine, University of Campania Luigi Vanvitelli, Via Luigi de Crecchio, 8, 80138 Naples, Italy; paola.stiuso@unicampania.it (P.S.); stefania.lama@unicampania.it (S.L.); antoniettagerarda.gravina@unicampania.it (A.G.G.); alessandro.federico@unicampania.it (A.F.); 4 Department of Advanced Medical and Surgical Sciences, University of Campania Luigi Vanvitelli, Via Luigi de Crecchio, 8, 80138 Naples, Italy; annalisa.itro@unicampania.it; 5 Department of Interdisciplinary Medicine, University of Medicine Aldo Moro, 70124 Bari, Italy; giannadipalma@tiscali.it (G.D.); francesco.inchingolo@uniba.it (F.I.)

**Keywords:** oral microbiota, oral dysbiosis, chronic gastritis, microbiome, *Fusobacterium nucleatum*, *Porphyromonas gingivalis*, *Candida albicans*, salivary markers, RT-PCR

## Abstract

Various bi-directional associations exist between oral health and gastro-intestinal diseases. The oral microbiome plays a role in the gastro-intestinal carcinogenesis and fusobacteria are the most investigated bacteria involved. This paper aims to review the current knowledge and report the preliminary data on salivary levels of *Fusobacterium nucleatum*, *Porphyromonas gingivalis* and *Candida albicans* in subjects with different gastro-intestinal conditions or pathologies, in order to determine any differences. The null hypothesis was “subjects with different gastro-intestinal diseases do not show significant differences in the composition of the oral microbiota”. Twenty-one subjects undergoing esophagastroduodenoscopy or colonscopy were recruited. For each subject, a salivary sample was collected before the endoscopy procedure, immediately stored at −20 °C and subsequently used for genomic bacterial DNA extraction by real-time PCR. Low levels of *F. nucleatum* and *P. gingivalis* were peculiar in the oral microbiota in subjects affected by *Helicobater pylori*-negative chronic gastritis without cancerization and future studies will elucidate this association. The level of *C. albicans* did not statistically differ among groups. This preliminary study could be used in the future, following further investigation, as a non-invasive method for the search of gastrointestinal diseases and associated markers.

## 1. Introduction

### 1.1. Oral Microbiota: An Overview

The mouth is the opening tract of the digestive system, and its unhealthy state has been bi-directionally associated with various systemic and gastro-intestinal diseases [1,2,3,4,5]. For example, atrophic glossitis and angular cheilitis may underlie Plummer–Vinson syndrome—a sideropenic dysphagia secondary to iron deficiency associated with gastric ulcerations [6] as well as pernicious anemia, which is due to the failure of the gastric cells to produce the intrinsic factor responsible for the absorption of vitamin B12 in the intestine [7]. Additionally, the oral manifestations of a number of chronic bowel diseases, such as Crohn’s and coeliac diseases, are well documented and oral signs and/or symptoms can be assumed to be related to these diseases [8,9,10].

The existence of an oral–gut axis has also been confirmed by the discovery of an association between intestinal bowel diseases (IBD) and pathogens of oral origin [11,12,13,14] as well as recent evidence that the administration of gut-derived probiotics can be useful in the prevention of dental caries [15]. This suggests that improving the condition of the gut microbiota may lead to a simultaneous improvement in the operational taxonomic units (OTUs) of bacteria residing in the oral cavity.

As defined by Berg et al. [16], the human microbiota is made up of all types of microorganisms (archaea, eukaryotes, bacteria and viruses), that live on and in the human body, each housed in specific ecological niches, including oral ones [17]. Instead, the term “microbiome” refers to all of their genomic material and products [18]. Ever since research into the oral microbiome began [19], new associations have been continuously identified between alterations in the composition of the oral microbiome and various gastrointestinal diseases. This is possible due to the development and use of high-throughput culture-independent technologies, such as reverse transcriptase-polymerase chain reaction (RT-PCR) [20] and next generation sequencing (NSG) [21], both capable of identifying microorganisms and their genes, even when they are not cultivable [22]. The “microbiome project” aims to identify the microbiome components of the entire human body [19] and the effects of various dysbiosis on human health. Approximately 700 species of prokaryotes have been identified in the oral microbiome. These species belong to 185 genera and 12 phyla, of which approximately 54% are officially named, 14% are unnamed (but cultivated) and 32% are known only as uncultivated phylotypes [9]. The 12 phlya are *Firmicutes*, *Fusobacteria*, *Proteobacteria*, *Actinobacteria*, *Bacteroidetes*, *Chlamydiae*, *Chloroflexi*, *Spirochaetes*, SR1, *Synergistetes*, *Saccharibacteria* (TM7) and *Gracilibacteria* (GN02) [23]. In addition, the oral cavity also contains diverse forms of microbes such as protozoa, fungi and viruses. *Entamoeba gingivalis* and *Trichomonas tenax* are the most commonly found protozoa and are mainly saprophytic. The *Candida* species is the most prevalent fungi seen associated with the oral cavity. Ghannoum et al. [24] carried out culture-independent studies on 20 healthy hosts and reported 85 fungal genera. The main species observed were those belonging to *Candida*, *Cladosporium*, *Aureobasidium*, *Saccharomycetales*, *Aspergillus*, *Fusarium* and *Cryptococcus* [25]. Of particular interest, *Candida albicans* is a dimorphic yeast that is occasionally found in healthy mouths as a saprophyte [26] but can infect oral mucosa after dysregulations of the normal oral flora, under local [27] and systemic circumstances, both para-physiological (pregnancies, elderly, early childhood) and iatrogenic (prolonged steroids and/or antibiotics therapies) [28] as well as in dysmetabolic/dysimmune pathologies (diabetes, obesity and/or immunodeficiencies) [29,30,31]. In all these situations, the antagonistic bacterial–fungal relationship favors the switch of *C. albicans* to its infectious phenotype, with carcinogenic potential, as has been reported for other microbial species such as papillomaviruses [32] in particular conditions [33].

**Figure 1:**
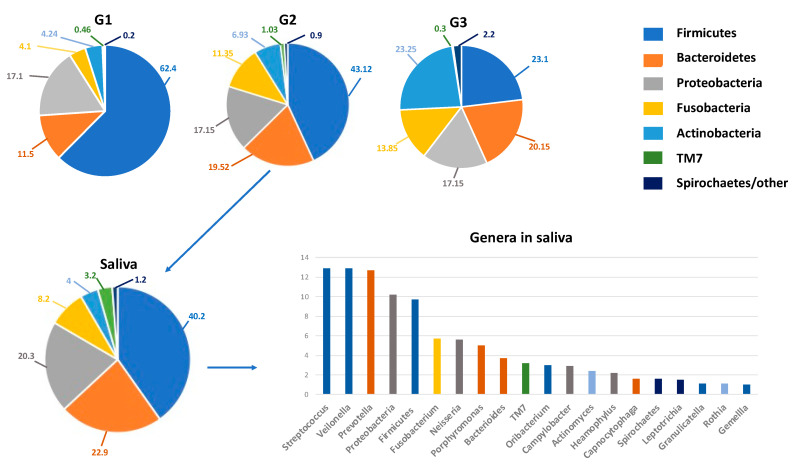
The oral microbiota of a healthy mouth. On **top**, the percentage composition of phyla in the various niches (G1–G3) of the oral microbiota. G1, Group 1: keratinized gingiva, hard palate and the buccal mucosa; G2, Group 2, tongue, tonsils, throat and saliva; G3, Group 3, sub- and supra-gingival plaque. On the **bottom**, details on the percentage composition of phyla (left) and genera mainly present in salivary microbiota (right). The genera are represented in descending percentages, and color bars reflect the phyla they belong to. The organisms inhabiting saliva account for 99.9% of all bacteria in the oral cavity and are usually planktonic organisms.

Under physiological conditions, the microorganisms of the core microbiota are qualitatively and quantitatively arranged in three different ecological niches/intraoral habitats as follows: Group 1 includes microorganisms at the level of the keratinized gingiva, the hard palate and the buccal mucosa; Group 2 comprises those on the tongue, tonsils, throat (posterior wall of the oropharynx) and in the saliva; and Group 3 comprises those in sub- and supra-gingival plaque [34,35] (Figure 1).

Oral dysbiosis consists of a qualitative and quantitative imbalance of the composition of the oral microbiota. The specific predominance of some pathogenic microorganisms over others is associated with specific oral and systemic diseases. *Fusobacterium nucleatum* and *Porphyromonas gingivalis* are the bacteria most frequently and variably investigated and are mainly known to be responsible for periodontal diseases, although they can also be found in subjects without periodontitis [36,37]. Scientific literature reports that their presence is associated with the onset and/or worsening of a wide range of systemic diseases [38], such as osteoporosis [5], cardiovascular diseases [39,40], rheumatoid arthritis [41] and neurodegenerative diseases such as Alzheimer’s and Parkinson’s [42,43].

### 1.2. Oral Dysbiosis and Gastrointestinal Diseases

The term “gastritis” refers to a series of acute or chronic inflammations of the stomach, secondary to endogenous or exogenous irritants, which determine a reparative and/or reactive response of the gastric mucosa. The etiology of acute gastritis (AC) recognizes numerous causes, such as drugs, caustic agents, radiations and traumas, while chronic gastritis (CG) is mainly sustained by *Helicobacter pylori* infections or autoimmunological triggers, and is dichotomized in *H. pylori*-related and *H. pylori*-unrelated gastritis [44]. The severity and persistence of inflammation in CG or the onset of ulcers, mainly related to *H. pylori* infections and non-steroidal anti-inflammatory drugs (NSAIDs), are associated with the risk of developing dysplasia of varying degrees and gastric cancers [45].

Furthermore, Cui et al. [46] analyzed the diversity of the oral microbiome of subjects with and without gastritis and found that the levels of 11 species decreased and 10 increased in patients with gastritis.

With regards to the gastro-intestinal system, *F. nucleatum* and *P. gingivalis* can reach the stomach directly during swallowing, but they and their toxins have also been found in the colon and in the fecal microbiome, because they pass gastric juices alive and by systemic dissemination from the ulcerated gingival pockets through the hematogenous route [47,48].

**Figure 2:**
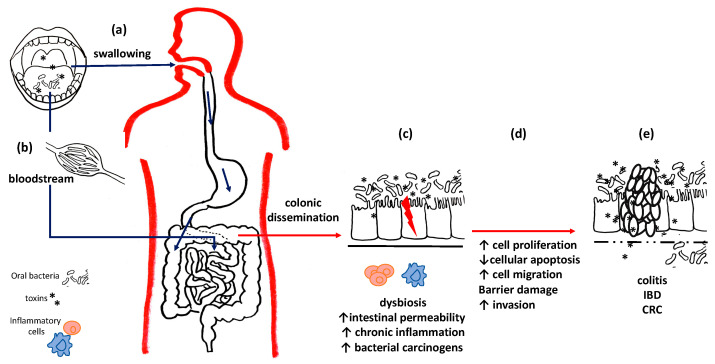
Oral bacteria and intestinal diseases. Oral bacteria can reach the intestines and stomach both through swallowing (**a**) and through the bloodstream (**b**). (**c**) At the level of the colon mucosa, they can compete with the local flora and establish intestinal dysbiosis. Pathogenic bacteria and their toxins alter the permeability of the basement membrane, perpetuate chronic inflammation and promote carcinogenesis. (**d**) Uncontrolled cell cycles reduced apoptotic efficiency and barrier damage results, thus leading to (**e**) chronic inflammatory diseases and the onset of cancers.

For these reasons, recent literature has focused on the hypothesis that gastric and colorectal cancers (CRC) and their precursors, such as gastritis and inflammatory bowel diseases (IBDs), may also be associated with oral dysbiosis [49,50]; furthermore, oral bacteria can colonize the gut microbiome, thus influencing intestinal and extra-intestinal health by altering the permeability of the intestinal mucosa and perpetuating chronic inflammatory states, both locally and systemically, with the hematogenous dissemination of lipopolysaccharides (LPSs) and other toxins responsible for distant consequences [51] as well as CRC and pancreatic cancers [49] (Figure 2).

Zhang et al. [52] suggested three mechanisms of action of oral microbiota in the pathogenesis of cancer. The first is related to the state of chronic inflammation induced, especially by anaerobic species in particular, such as *Porphyromonas, Prevotella* and *Fusobacterium*. These pathogens are, in fact, able to stimulate the production of important mediators of the inflammatory process, such as the cytokines interleukin (IL)-1β, IL-6, IL-17, IL-23, and Tumor Necrosis Factor α (TNF-α). The consequent harmful effects develop mainly on fibroblasts, epithelial and endothelial cells, and components of the extracellular matrix, with an increase in the expression of the metalloproteases MMP-8 and MMP-9 and a consequent increase in cell proliferation, mutagenesis, initiation of angiogenic processes and oncogenesis [53,54].

A second mechanism of action involves the ability of oral bacteria to influence cell proliferation, rearrangement of the cytoskeleton, activation of the transcription factor NF-kB and inhibition of apoptosis. For example, it has been shown that *P. gingivalis* can inhibit apoptosis by influencing different pathways, reducing the expression levels of proapoptotic molecules such as p53 [54,55] and Bad [54,56], inhibiting the activation of caspase-9 [55] and increasing the production of anti-apoptotic factors, such as Bcl-2 [54,57].

The third mechanism involves the oral pathogens’ production of many carcinogenic substances. These include reactive oxygen (ROS) and nitrogen (RNS) species [58,59], mainly produced by species such as *Streptococcus oralis*, *S. mitis*, *S. sanguinis*, *S. gordonii*, *S. oligofermentans* [60], *Lactobacillus fermentum*, *L. jensenii*, *L. acidophilus*, *L. minutus* and *Bifidobacterium adolescentis* [61]. ROS and RNS production induces NADPH oxidase and nitric oxide synthase (NOS), respectively, along with their reactive oxygen and nitrogen species, which have been identified in various tumor types [62,63].

Other species, such as *Porphyromonas gingivalis*, *Prevotella intermedia*, *Aggregatibacter actinomycetemcomitans* and *Fusobacterium nucleatum*, produce volatile sulphur compounds (VSC), including hydrogen sulphide (H2S), methyl mercaptan (CH3SH), dimethyl sulphide ((CH3) 2S), (CH3SS), (CH3SS), and (CH3SS), whose presence is often associated with the onset of cancer [64,65,66].

Some species are able to produce more acids (e.g., *Peptostreptococcus stomatis* aciduric produces acetic, butyric, isobutyric, isovaleric, and isocaproic acids) [67] that can add to the acidic and hypoxic microenvironment of tumors, thereby increasing metastatic efficiency [68,69]. Furthermore, various oral microbial species, such as *S. gordonii*, *S. mitis*, *S. oralis*, *S. salivarius* and *S. sanguinis* streptococci [70], metabolize alcohol to acetaldehyde, which is indisputably carcinogenic.

Cordero et al. [71] reported that the relationship between oral hygiene and intestinal inflammation, which are mutually involved through signaling pathways, are linked to tumor-promoting inflammation. In fact, during the inflammatory process, the massive presence of pathogens or the simple imbalance of the oral microbiota play an important role in the onset of CRC from a chronic inflamed bowel, such as in cases of IBD. The authors based this hypothesis on the evidence that part of the gut microbiota comes from the oral one. Thus, as stated by Flemer et al. [49], “the oral microbiota in colorectal cancer is distinctive and predictive”. After profiling the microbiota from oral swabs, colonic mucosae and stools in individuals with CRC, colorectal polyps and healthy controls, they concluded that (i) oral bacteria were more abundant in CRC than in healthy controls, (ii) that the oral microbiome of healthy controls was different from those with CRC, and (iii) that *F. nucleatum* was most abundant in CRCs.

Numerous studies have also reported a significant increase in *F. nucleatum* in the gastric microbiome of subjects with gastric cancers and gastritis.

In 2013, Salazar et al. [72] conducted a clinical study to measure the levels of periodontal pathogenic bacteria in dental plaque and salivary samples from subjects with gastric precancerous lesions via quantitative RT-PCR. They found a high but not statistically significant increase in *P. gingivalis* and therefore, they hypothesized that high levels of periodontal colonization by pathogens may be associated with an increased risk of precancerous gastric lesions. In 2017, Coker et al. [73] identified differences in microbial diversity and richness between gastric cancers and various types of gastritis, thus indicating the presence of microbial dysbiosis in gastric carcinogenesis. Specifically, *Prevotella intermedia* and *F. nucleatum*, together with *Prevotella oris* and *Catonella morbi*, were significantly enriched in the gastric cancer microbiome compared to precancerous stages and they formed an increasingly strong co-occurrence network with disease progression.

The recent works of Yamamura et al. [74] and Hsieh et al. [75] have reinforced these correlations. Yamamura et al. [74] detected a significant increase in the mount of *F. nucleatum* DNA in oesophageal and gastric cancers as well as CRCs. Hsieh et al. [75] profiled gastric bacterial species in patients with gastritis and gastric cancer and found that *F. nucleatum*, along with *Clostridium colicans*, was frequently abundant in gastric cancer patients, supporting a specific gastric cancer signature.

Regarding *C. albicans*, the scientific literature has so far paid little attention to the study of its relationship with systemic health, limited to the role of candidiasis in a few systemic diseases, and has not yet considered its possible gastro-intestinal implications [76,77]. The main findings on oral bacteria associated with gastrointestinal disease are reported in Table 1.

In the light of the above review of the literature, the aim of this study was to establish a possible association of the salivary levels of *F. nucleatum*, *P. gingivalis* and *C. albicans* with various gastro-intestinal conditions and/or pathologies, in order to highlight any differences and their possible clinical significance and correlations. The null hypothesis was “subjects with different gastro-intestinal diseases do not show significant differences in the composition of the oral microbiota”.

## 2. Materials and Methods

### 2.1. Patients

All the procedures in the present study involving human participants were performed after approval from the Internal Ethics Committee (protocol number #68/2020, Comitato Etico Università della Campania “Luigi Vanvitelli”—Azienda Ospedaliera Universitaria “Luigi Vanvitelli”—AORN “Ospedale dei Colli”), and in accordance with the 1964 Helsinki declaration and its later amendments.

A series of consecutive subjects referred to the Digestive Endoscopy Unit of the University of Campania “Luigi Vanvitelli”, Naples, Italy, were considered. The exclusion criteria were as follows: recent antimicrobial therapy and/or use of oral antiseptic (less than two weeks prior the enrollment) and presence of chronic and/or acute confounding infections, such as HCV, HBV and HIV, established by serological tests exhibited by each patient invited to participate. All the subjects who agreed to participate, gave their informed written consent for their anamnestic data and a salivary sample to be collected before the scheduled endoscopic procedure, which was performed in accordance with the standards protocols.

For subjects with clinical suspicion of gastric diseases, a complete esophagastroduodenoscopy (EGDS) was performed, while patients with suspected CRC or a past history of CRC underwent colonoscopy (CS) for post-cancer follow-up. When necessary, one or more biopsies were performed simultaneously with the endoscopic procedure to analyze suspicious lesions with a conventional histology.

Two healthy subjects, who underwent CS for hemorrhoids and had no pathological findings or other gastrointestinal symptoms, were considered as the control group.

Saliva was collected by asking the patient to spit once per minute into a sterile Eppendorf, mainly two hours after the last brushing of teeth in the morning and prior to the endoscopic procedure, until the appropriate amount (5 mL) was obtained. The study was double-blinded—both the examiner collecting the saliva and microbiologist dealing with the samples were blinded to the gastro-intestinal conditions of the patients.

### 2.2. Saliva Analysis for Microbiota Evaluation

Salivary samples frozen at −20 degree Celsius were used for genomic bacterial DNA extraction with the Qiaamp DNA mini kit (Qiagen, Germantown, MD, USA) according to the manufacturer’s instructions.

Real-time PCR was carried out to detect the presence of periodontal pathogens with the LC FastStart DNA Master SYBR Green kit (Roche Diagnostics, Penzberg, Germany) in a 20 μL final volume using 2 μL of DNA, 3 mM MgCl_2_, and 0.5 mM sense and antisense primers (Table 2).

After amplification, the melting curve analysis was performed by heating to 95 °C for 15 s with a temperature transition rate of 20 °C s^−1^, cooling to 60 °C for 15 s with a temperature transition rate of 20 °C s^−1^, and then heating the sample at 0.1 °C s^−1^ to 95 °C. The results were then analyzed using the LightCycler software (Roche Diagnostics, Penzberg, Germany) [78,79].

The standard curve of each primer pair was established with serial dilutions of the DNA; all PCR reactions were run in triplicate.

### 2.3. Statistical Analysis

Significant differences among the groups were assessed using the t-student test and Excel (ver.16.16^®^ 2018 Microsoft). The data were expressed as means ± standard deviation (SD) of three independent experiments.

## 3. Results

Overall, 21 subjects were considered (13 women, eight men; mean age 58.86 ± 13.49 years). All seven subjects with CG were negative for *H. pylori*; the six ex-CRC subjects and the four healthy controls did not report any pathological findings. Four subjects reported histological findings of CRC.

The mean levels of *F. nucleatum*, *P. gingivalis* and *C. albicans* in each patient and in each group are detailed in Table 3. With regard to *C. albicans*, it was discontinuously found and statistical differences were not reported, neither between groups (Table 3), nor between subjects with (*n* = 5; mean levels of *C. albicans*: 82.9 ± 156.86) and without removable dentures (*n* = 16; mean levels of *C. albicans*: 20.00 ± 51.00); hence, its analysis was excluded from further examinations. Moreover, no differences were found in *F. nucleatum* and *P. gingivalis* amounts between denture wearers and non-wearers.

The levels of *F. nucleatum* were statistically the lowest in the CG group compared to any other group, while, unexpectedly, they were significantly higher in the control group than in the CRC group, and no significant differences were reported between the healthy subjects and the ex-CRC patients (Table 4).

The lowest levels of *P. gingivalis* were found in the CG group with a statistically significant difference as compared to the healthy controls. The latter group showed higher but not statistically relevant amounts of *P. gingivalis* compared to the CRC and ex-CRC subjects, and the CRC group showed higher mean amounts than the CRC group (Table 5).

## 4. Discussion and Conclusions

Periodontitis is a biofilm-induced chronic condition which involves inflammation and destruction of periodontal tissue [80] by oral bacteria and is considered a global disease burden [81], being sixth among the most prevalent human diseases [82]. Many studies in the last 20 years have shown the existence of a clear association between periodontitis and the onset of other chronic systemic inflammatory diseases [83] due to the inflammatory state and activation of the immune response triggered by periodontal pathogens, following the onset of oral microbiota dysbiosis [84].

Diseases associated with periodontitis include diabetes [85], head and neck cancer [86], pulmonary disease [87], survival of dental implants [88] and cardiovascular diseases [89]. In recent years, there has been a growing interest in the existence of an oral–gut axis and its related pathologies. Yu et al. reported a significantly positive association between peptic ulcer and periodontal disease [90]. The presence of periodontal disease has often been detected in patients with IBD [91], and it has also been shown, conversely, that patients with IBD suffer from a more severe degree of periodontal disease [92]. Wei et al. [93] reported that chronic periodontitis (CP) was potentially correlated with oral *H*. *pylori* in adults, and that it may be a possible risk factor for CP. Boylan et al. and Byun et al. showed an increased risk of gastric and duodenal ulcer among patients with periodontal disease [94,95], while Umeda et al. suggested that patients with periodontitis who harbor *H. pylori* in the oral cavity should be closely monitored [96]. However, the pathway underlying the correlation between periodontitis and *H. pylori*-related chronic gastritis/peptic ulcer is not completely understood and needs to be studied more thoroughly.

Another study stated that the salivary microbiota can affect the development of the intestinal microbiota, as saliva flows through the gastrointestinal tract, allowing the bacteria present in it to easily reach the intestine. It has in fact been shown, through a study aimed at assessing the metatranscriptome and metagenome of the human gut microbiota, that the DNA of bacteria belonging to the salivary microbiota is detectable in the gut even in low concentrations [97].

This review provides preliminary data on the assessment of salivary levels of *F. nucleatum*, *P. gingivalis* and *C. albicans* in patients with CG and with a history of CRC.

Twenty-one subjects were enrolled: nine had undergone EGDS and 12 CS. In each subject the levels of *F. nucleatum*, *P. gingivalis* and *C. albicans* were measured by RT-PCR and correlated with their endoscopic and histologic diagnosis, to establish any differences between the groups.

*C. albicans* was found intermittently, and no statistical differences were reported either between groups or between subjects with and without removable dentures (as well as the amounts of *F. nucleatum* and *P. gingivalis*). The finding of *C. albicans* was not associated with clinical signs of oral candidiasis, thus suggesting a carrier state of some subjects, without any correlation with their gastrointestinal conditions.

Levels of *F. nucleatum* were the lowest in the CG group and the highest in the control group. The ex-CRC patients showed relatively, but not significantly, higher levels than those of CRC group. This last finding contradicted the literature reporting the increase of *F. nucleatum* in stool samples from CRC subjects and its association with colon carcinogenesis and chronic cancer-related inflammation [48]. If we hypothesize that these marked differences may be related to the source of the sample (saliva instead of feces), it would be reasonable to exclude salivary tests for CRC screening, but further comparative studies should clarify this point better.

The lowest levels of *F. nucleatum* and *P. gingivalis* were found in the CG group, with statistically significant differences in *F. nucleatum* compared to each group, and *P. gingivalis,* as compared to healthy subjects. In contrast, subjects with ex-CRC revealed a different profile in which there were relative high concentrations of *F. nucleatum* and low *P. gingivalis*, as compared to patients with CRC.

Another key feature of the CG group was that all subjects were *H. pylori*-negative. Literature reports that *H. pylori*-positive individuals have a significant increase in the amount of *F. nucleatum* in the oral cavity, as *H. pylori* selectively adheres and co-aggregates with *Fusobacteria* [98]. Therefore, it is reasonable to speculate that low levels of *F. nucleatum* can influence or be influenced by the lack of *H. pylori*. What is still unclear is whether *H. pylori*-negativity should be considered a consequence of low levels of *F. nucleatum*, or whether the low levels of *F. nucleatum* are due to a lack of *H. pylori*.

In the first case, the expression of *H. pylori* could be considered to be directly correlated to the quantity of *F. nucleatum* and, therefore, it could be hypothesized as indirectly reducing *H. pylori* by acting on the salivary reduction of *F. nucleatum* with oral hygiene protocols to directly rebalance the composition of the oral microbiota and, indirectly, the gastric one. This intervention could reduce the need for antibiotic therapies for the eradication of *H. pylori* by abolishing their adverse effects and drug-resistances. Conversely, if the low levels of *F. nucleatum* were a consequence of the lack of *H. pylori*, it would be possible to indirectly estimate the presence/absence of *H. pylori* in CG by measuring the amount of *F. nucleatum* in the oral cavity. In any case, although these doubts are still to be clarified, and these hypotheses require confirmation through larger clinical studies, the hypothesis of considering the measurement of *F. nucleatum* as a predictor of the presence of *H. pylori* in CGs and their cancerization has been corroborated by various studies [98,99,100,101].

Furthermore, the microbial diversity in subjects with gastritis *H. pylori*-negative compared with those with gastritis *H. pylori*-positive, was proven by several studies [101] as well the fact that saliva and stomach aspirates share similar bacterial composition and significantly highest abundance of Fusobacteria, compared to other gastro-intestinal sites. Particularly, Fusobacteria were found to be more abundant in the stomach than saliva in a series of subjects with gastritis *H. pylori*-negative [102]. On this basis, it is reasonable and follows Zhao et al. [102] that, if the saliva is the main source for the gastric microbiome, a correlation between *H. pylori* and oral bacterial species may exist and may influence and/or be influenced by each other [102].

The consistency of this study was strongly affected by the small sample size; thus, it must be considered as just “exploratory” and in need of improvement. Unfortunately, this was due to the interruption of recruitment after the outbreak of the COVID-19 pandemic, in March 2020.

The intestinal and oral environments are infinitely complex, and the microbiota of these environments is a key element in maintaining homeostasis, so saliva should be considered as a means of monitoring the intestine for future research in gastrointestinal tract diseases.

However, the preliminary results encourage and recommend further cohort studies on patients suffering from CG, in order to establish whether the salivary quantification of *F. nucleatum* and *P. gingivalis* can actually serve as a non-invasive marker for monitoring the onset of *H. pylori* or cancerization.

## Figures and Tables

**Table 1:** Oral bacteria associated with gastrointestinal diseases.

G-i Diseases	Oral Bacteria/Fungi	Main Findings	Ref.
Gastritis	*Streptococci*, *Fusobacteria*	significantly higher in gastritis vs. healthy controls	[46]
	*Veillonella parvula*, *Corynebacterium matruchotii*, *Kingella oralis*, *Atopobium rimae*, *Aggregatibacter aphrophilus*, *Streptococcus sanguinis*, *Acinetobacter lwoffii*, *Prevotella amnii*, *Prevotella bivia*, *Cardiobacterium hominis* and *Oribacterium sinus*	decreased in gastritis patients vs. healthy control	[46]
	*Streptococcus infantis*, *Treponema vincentii*, *Leptotrichia unclassified*, *Campylobacter rectus*, *Campylobacter showae*, *Capnocytophaga gingivalis*, *Leptotrichia buccalis*, *Campylobacter concisus*, *Selenomonas flueggei* and *Leptotrichia hofstadii*	increased in gastritis patients vs. healthy control (mainly *Campylobacter* spp.)	[46]
Gastric Precancerous lesions	*Campylobacter concisus*	positively associated with the precancerous cascade of gastritis	[46]
	*Porphyromonas gingivalis*, *Treponema denticola*	increased in dental plaque of subjects with gastric precancerous lesions	[72]
	*Actinobacillus actinomycetemcomitans*, *Treponema denticola*	significantly associated with gastric precancerous lesions	[72]
	*Tannerella forsythia*	significantly inversely associated with gastric precancerous lesions	[72]
Oesophageal and Gastric Cancers	*Tannerella forsythia*, *Porphyromonas gingivalis*	associated with higher risk of oesophageal cancers	[52]
	*Streptococcus anginosus*	higher in oesophageal cancer tissues than in oral cancer tissues	[52]
	*Fusobacterium nucleatum*	higher in oesophageal cancer tissues than matched normal mucosa; significantly associated with tumor stage and cancer-specific survival	[52]
	*Neisseria* spp., *Candida glabrata*	potential role in alcohol-related carcinogenesis	[52]
	*Parvimonas micra*, *Peptostreptococcus stomatis*, *Prevotella intermedia*, *Fusobacterium nucleatum*, *Prevotella oris*, *Gemella* and *Catonella morbi*, *Streptococcus anginosus*, *Dialister pneumosintes*, *Slackia exigua*	significantly increased in gastric cancer compared with precancerous stages	[73]
Inflammatory bowel diseases (IBDs)	*Bacteroidetes*	significantly increased in IBDs	[51]
	*Proteobacteria* and *Actinobacteria*	increased in IBDs	[51]
	*Campylobacter concisus*	increases the mucosal permeability by affecting the tight junctions in IBDs	[51]
	*Fusobacterium nucleatum*	overrepresented in IBDs	[51]
	*Candida albicans*	isolated from the intestine more frequently in IBD patients	[51]
CRC	*Haemophilus* spp., *Prevotella* spp., *Alloprevotella* *Lachnoanaerobaculum*, *Neisseria* and *Streptococcus* spp.	less abundant in CRC than healthy controls	[49]
	*Fusobacterium nucleatum*, *Parvimonas micra*, *Peptostreptococcus stomatis*, *Dialister pneumosintes*	tumor-associated bacteria	[49]
	*Peptostreptococcus*, *Parvimonas*, *Fusobacterium*	more abundant in CRC than in healthy controls	[49]
	*Fusobacterium nucleatum*	induces inflammatory response and promotes CRC development	[52]
	*Treponema denticola*, *Prevotella intermedia*	increases the CRC risk	[52]
	*Porphyromonas gingivalis*	causes inflammation and promotes tissue degenerative processes	[52]
	*Fusobacterium nucleatum*	associated with CRC regional lymph node metastases	[55]
	*Fusobacterium nucleatum*, *Selenomonas*, *Prevotella*, *Parvimonas micra*, *Peptostreptococcus stomatis*	increased in CRC; induces colon cancer growth and progression	[71]
	*Lachnospiraceae*	can protect against CRC	[71]
	*Fusobacterium nucleatum*	sustains both the biofilm and the CRC tumorigenesis	[47]

**Table 2:** Primer sequence and amplification conditions.

Gene	Primers Sequence	Conditions	ProductSize (bp)
*Fusobacterium nucleatum*	5′-AGAGTTTGATCCTGGCTCAG-3′ 5′-GTCATCGTGCACACAGAATTGCTG-3′	5″ at 95 °C, 16″ at 55 °C, 8″ at 72 °C for 40 cycles	407
*Porphyromonas gingivalis*	5′-TGTAGATGACTGATGGTGAAAACC-3′ 5′-ACGTCATCCCCACCTTCCTC-3′	5″ at 95 °C, 5” at 52 °C, 4″ at 72 °C for 40 cycles	198
*Candida albicans*	5′-TTTATCAACTTGTCACACCAGA-3′ 5′-GGTCAAAGTTTGAAGATATACGT-3′	10″ at 95 °C, 10″ at 58 °C, 15″ at 72 °C for 30 cycles	354

**Table 3:** Datasets of the subjects enrolled.

	Id. Patient	Age (Years)	Sex	F.n. (ng/dL)	P.g. (pg/mL)	C.a. (pg/mL)	F.n. per Group (Mean ± SD)	P.g. per Group (Mean ± SD)	C.a. per Group * (Mean ± SD)
CG Group	2	49	F	0.10	0.03	156	1.10 ± 1.62	0.57 ± 1.17	39.36 ± 57.80
	3	50	F	0.02	0.05	0			
	6	68	F	2.40	0.04	0			
	8	23	F	4.20	3.20	71.5			
	10	47	F	0.00	0.15	12			
	30	55	F	0.93	0.08	0			
	44	46	F	0.08	0.44	36			
Ex-CRC Group	17	67	M	40.50	29.75	0	31.62 ± 34.40	7.78 ± 11.37	14.08 ± 15.76
	22	58	M	3.85	1.4	0			
	24	66	F	1.37	8.75	0			
	27	63	M	95.00	0.01	32.5			
	48	71	F	28.50	6.75	22.5			
	50	62	M	20.50	0.000	29.50			
CRC Group	20	80	M	9.50	0.05	365.5	9.13 ± 6.03	2.88 ± 3.68	91.25 ± 182.50
	29	63	M	1.50	0.03	0			
	31	87	F	9.25	3.70	0			
	39	63	M	16.25	7.75	0			
Healthy control	9	49	F	56.50	296.50	0	65.06 ± 14.92	110.19 ± 127.37	0
	42	54	F	85.00	10.25	0			
	45	51	M	67.50	78.00	0			
	34	64	F	51.25	56.5	0			

F.n. *Fusobacterium nucleatum*; P.g. *Porphyromonas gingivalis*; C.a. *Candida albicans*. * t-student test revealed no significant differences between any paired groups.

**Table 4:** Correlations between groups: *F. nucleatum* levels.

	CG Group	Ex-CRC Group	CRC Group	Healthy Group
Sample size	7	6	4	4
Mean F.n. values	1.10	31.62	9.13	65.06
Standard Deviation	1.62	34.40	6.03	14.92
t-student test			CG vs. ex-CRC * *p* < 0.05 CG vs. CRC * *p* < 0.05 CG vs. Healthy * *p* < 0.05 Healthy vs. CRC * *p* < 0.05 Healthy vs. Ex-CRC n.s. (*p* = 0.11) CRC vs. ex-CRC n.s. (*p* = 0.24)	

* Statistically significant at *p* < 0.05; n.s.: Not Significant.

**Table 5:** Correlations between groups: *P. gingivalis* levels.

	CG Group	Ex-CRC Group	CRC Group	Healthy Control
Sample size	7	6	4	4
Mean P.g. values	0.57	7.78	2.88	110.19
Standard Deviation	1.17	11.37	3.68	127.37
t-student test			CG vs. ex-CRC n.s. (*p* = 0.12) CG vs. CRC n.s. (*p* = 0.15) CG vs. Healthy * *p* < 0.05 Healthy vs. CRC n.s. (*p* = 0.14) Healthy vs. Ex-CRC n.s. (*p* = 0.07) CRC vs. ex-CRC n.s. (*p* = 0.44)	

* Statistically significant at *p* < 0.05; n.s.: Not Significant.
